# Dairy Products Consumption and Risk of Type 2 Diabetes: Systematic Review and Dose-Response Meta-Analysis

**DOI:** 10.1371/journal.pone.0073965

**Published:** 2013-09-27

**Authors:** Dengfeng Gao, Ning Ning, Congxia Wang, Yuhuan Wang, Qing Li, Zhe Meng, Yang Liu, Qiang Li

**Affiliations:** 1 Department of Cardiology, The Second Affiliated Hospital, Xi'an Jiaotong University School of Medicine, Xi'an, Shaanxi, P.R. China; 2 Key Laboratory of Environment and Genes Related to Diseases (Xi'an Jiaotong University), Ministry of Education, Xi'an, Shaanxi, P. R. China; 3 Department of Endocrinology, The Second Affiliated Hospital, Xi'an Jiaotong University School of Medicine,Xi'an, Shaanxi, P.R. China; 4 Department of Epidemiology, Xi'an Jiaotong University School of Medicine, Xi'an, Shaanxi, P.R. China; Iran University of Medical Sciences, Iran (Republic of Islamic)

## Abstract

**Background:**

The consumption of dairy products may influence the risk of type 2 diabetes mellitus (T2DM), but inconsistent findings have been reported. Moreover, large variation in the types of dairy intake has not yet been fully explored.

**Methods and Results:**

We conducted a systematic review and meta-analysis to clarify the dose–response association of dairy products intake and T2DM risk. We searched PubMed, EMBASE and Scopus for studies of dairy products intake and T2DM risk published up to the end of October 2012. Random-effects models were used to estimate summary relative risk (RR) statistics. Dose-response relations were evaluated using data from different dairy products in each study. We included 14 articles of cohort studies that reported RR estimates and 95% confidence intervals (95% CIs) of T2DM with dairy products intake. We found an inverse linear association of consumption of total dairy products (13 studies), low-fat dairy products (8 studies), cheese (7 studies) and yogurt (7 studies) and risk of T2DM. The pooled RRs were 0.94 (95% CI 0.91–0.97) and 0.88 (0.84–0.93) for 200 g/day total and low-fat dairy consumption, respectively. The pooled RRs were 0.80 (0.69–0.93) and 0.91 (0.82–1.00) for 30 g/d cheese and 50 g/d yogurt consumption, respectively. We also found a nonlinear association of total and low-fat dairy intake and T2DM risk, and the inverse association appeared to be strongest within 200 g/d intake.

**Conclusion:**

A modest increase in daily intake of dairy products such as low fat dairy, cheese and yogurt may contribute to the prevention of T2DM, which needs confirmation in randomized controlled trials.

## Introduction

The prevalence of type 2 diabetes mellitus (T2DM) is a growing public-health burden worldwide, particularly in developing countries. The prevalence of T2DM is estimated to reach 552 million worldwide by 2030 [1]. T2DM may cause substantial morbidity and mortality and is associated with enormous economic, health, and societal costs [2], [3]. Moreover, as compared with unaffected people, those with T2DM are at increased risk of other chronic illnesses, including cardiovascular disease; T2DM more than doubles the risk of a heart attack or stroke [4], [5]. Therefore, the identification of modifiable risk factors for primary prevention of T2DM is of considerable public health importance.

T2DM has genetic components but is also directly influenced by modifiable lifestyle factors, including dietary behaviors [6]. Dairy consumption might affect T2DM. Experimental studies indicated that dairy protein, such as whey protein, has insulinotropic and glucose-lowering properties [7].The Multi-Ethnic Study Atherosclerosis [8] and Cardiovascular Health study [9] suggested that fatty acids in dairy might be responsible for lower risk of T2DM. Epidemiological studies of dairy products and T2DM risk have given mixed results [10], [11], [12], [13], [14], [15], [16], [17], [18], [19], [20], [21], [22], [23]. Some cohort studies have reported inverse associations of intake of total and low-fat dairy products, milk and/or yogurt and T2DM risk, but other studies found no association [10], [11], [19], [20], [23]. One meta-analysis of 7 studies reported a significant inverse association of dairy intake and risk of T2DM [24]. However, the large variation in types of dairy consumed has not been fully explored. Furthermore, the dose–response relationship needs to be clarified as well as any gender or geographic differences in the T2DM risk. In addition, possible confounding by other lifestyle factors needs to be explored to firmly establish the potential preventive role of dairy products in T2DM.

We conducted a meta-analysis of population-based cohort studies to investigate dose–response associations of consumption of total, low-fat, and full-fat dairy products as well as different types of dairy products and risk of T2DM.

## Methods

### Data Sources and Search Strategy

We followed standard criteria for conducting and reporting meta-analyses of observational studies (MOOSE). Two authors (DG and NN) independently did a literature search MEDLINE via PubMed (published from 1966 to March 2013), EMBASE (published from 1980 to March 2013), and Scopus (www.scopus.com) with no restriction on language. To identify studies of milk or dairy product intake and T2DM risk, we used both the medical subject heading (MeSH) terms (“Diabetes Mellitus” AND (milk OR dairy)) and searched the text using the terms (‘diabetes’/exp OR diabetes') AND (‘dairy’/exp OR dairy OR ‘milk’/exp OR milk). We also searched the reference lists of all studies retrieved and published systematic reviews and meta-analysis.

### Study Selection

All abstracts retrieved were examined independently by 2 investigators (DG and NN) who then retrieved the full text of potential articles. Disagreements were resolved by consensus, and if necessary, with a third author (CW). We included prospective cohort studies and case-cohort studies assessing the association of consumption of total dairy products or specific types of dairy products and T2DM. To be included in the analyses, articles needed to contain estimates of the relative risk (RR) (such as odds ratios [ORs], hazard ratios [HRs] or risk ratios) with 95% confidence intervals (95% CIs). We excluded animal studies, clinical trials, cross sectional studies, case-control studies, and studies that examined other associations. For the dose–response analysis, a quantitative measure of intake had to be provided. If the article lacked data, we attempted to contact the author.

### Data Extraction and Quality Assessment

We extracted the following data from each study: country where the study was conducted, follow-up period, sample size, gender, age, number of cases, dietary assessment method (type, number of food items and whether the food intake had been validated), type of dairy product (e.g., total dairy, milk, cheese), quantity of intake, HRs, RR values, and ORs and 95% CIs for dairy product intake and, when available, the number of cases and participants or person-years for each category of dairy product consumption. Two authors (YL and ZM) independently performed the data extraction. Any disagreements were resolved by discussion.

Two independent reviewers (DG and NN) evaluated the quality of the selected studies by using a modified scoring system that was based on a recently used system (designed with reference to QUATSO [25], MOOSE [26], and STROBE [27]) that allowed for a total score of 0 to 6 points (6 indicating highest quality) [28]. The system allocates one point each for 1) any justification given for the cohort; 2) appropriate inclusion and exclusion criteria used; 3) outcome (diagnosis of T2DM not solely based on self-reporting); 4) intervention (participants' usual dairy consumption assessed with a validated tool); 5) statistical analysis (adjustments made for age, sex, body mass index, and family history of T2DM, total energy intake and physical activity, these being proven risk factors for type 2 diabetes); and 6) any other adjustments performed (such as glycemic load and dietary factors).

### Statistical analysis

HRs and RRs were assumed to be approximately the same measure of relative risk. For articles reporting ORs, we estimated the RRs from the ORs using a previously published correction method [29]. To take into account heterogeneity between studies, we used a random-effects models to calculate summary RRs and 95% CIs for the highest versus lowest level of dairy product intake and for the dose–response analysis. The natural logarithm of the RR from each study was weighted by the inverse of its variance and pooled across studies. A two-tailed P<0.05 was considered statistically significant. Articles that reported findings for men and women separately were considered 2 studies when the observed items were combined.

For the dose–response analysis, we used GLST command in Stata software as the method proposed by Greenland and Longnecker [30] and Orsini et al. [31] to compute study-specific slopes (linear trends) and 95% CIs from the natural logs of the RRs and 95% CIs across categories of dairy product intake.

For each study, the median or mean level of dairy product intake for each category was assigned to each corresponding RR. When the median or mean intake per category was not provided, we assigned the midpoint of upper and lower boundaries in each category as the average intake. If the highest or the lowest category was open-ended, we assumed that the open-ended interval length had the same length as the adjacent interval. If the intake was reported in densities (i.e., per 1000 kcal), we recalculated the reported intake as absolute intake using the mean or median energy intake reported in the article [14]. When studies reported the intake in servings and times per day or week, we converted the intake to grams of intake per day using standard units of 244 g (or 244 ml) for milk, 43 g for cheese (2 slices) and 177 g for total dairy products from the serving sizes reported in the *US Department of Agriculture Food and Nutrient Database for Dietary Studies* [32]. Pooled estimates were expressed in rounded numbers that approximated a normal portion size and fitted within the range of dairy intake of all studies (i.e., 200 g for milk and total, low-fat, and full-fat dairy; 50 g for yogurt; and 30 g for cheese).

To examine a potential nonlinear association between dairy products intake and T2DM risk, we performed a 2-stage, random-effects, dose-response meta-analysis, as recently summarized [33]. In the first stage, we constructed study-specific restricted cubic spline models, with 4 knots at fixed percentiles (5%, 35%, 65%, 95%) of the exposure distribution by using generalized least-squares regression. In the second stage, we combined the 2 regression coefficients and the variance/covariance matrix that had been estimated within each study, using the restricted maximum likelihood method in a multivariate random-effects meta-analysis. The pooled relative risks for specific exposure values were then estimated. A P value for nonlinearity was calculated by testing the null hypothesis that the coefficient of the second spline was equal to zero.

Heterogeneity among studies was assessed by I^2^, the amount of total variation explained by the between-study variation, and the Q test. We conducted subgroup and random effects univariate and multivariate meta-regression to investigate potential sources of heterogeneity we performed for the primary outcomes. Publication bias was assessed with funnel plots, Begg's test and Egger's test. Stata v12.0 (Stata Corp, College Station, TX) was used for all the statistical analysis.

## Results

### Study characteristics

**Figure 1:**
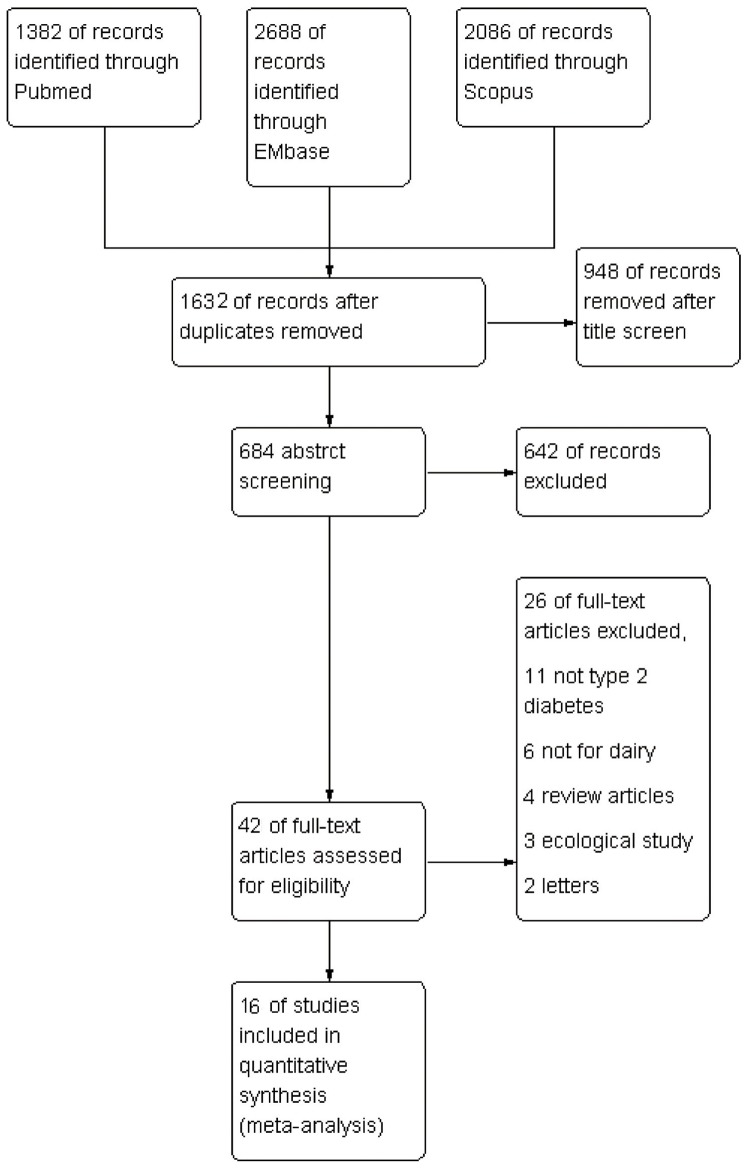
Flow chart for the selection of studies for meta-analysis of the association of dairy products intake and type 2 diabetes (T2DM).

**Figure 2:**
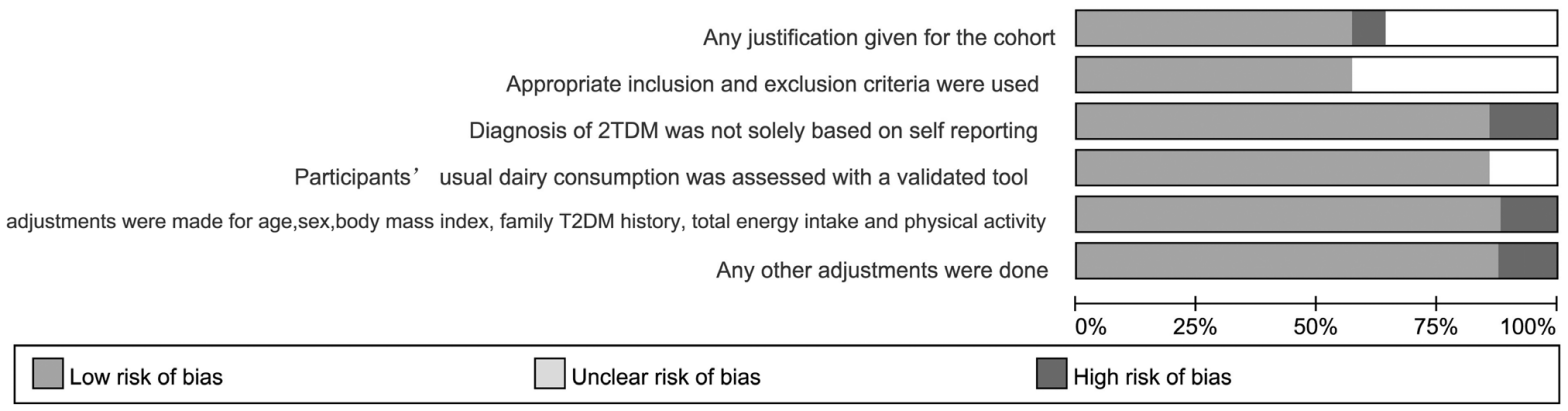
Methodological quality across included studies.

We included 15 prospective cohort studies and 1 case–cohort study in our analysis (Figure 1). 6 of the studies [12], [13], [14], [15], [16], [18] were performed in the United States, 6 in Europe [11], [19], [21], [23], [34], [35], 2 in Asia [17], [22] and 2 in Australia [10], [20]. The articles were published between 2005 and 2013 and included 526,998 subjects (including 29,789 T2DM cases). Characteristics of included studies are in Table 1. Figures 2 show assessments by risk of bias. The studies were generally of moderate quality. More than 75% of the studies met 4 of the quality items as reported and 8 studies met all requirements. Interrater reliability for assessing quality items was good (κ = 0.86, P<0.01).

**Table 1:** Characteristics of the cohort studies of dairy products intake and type 2 diabetes mellitus (T2DM).

Author,y	populati	Country	Men	Age, y	Follow	Subjects	Dietary	Dairy quantity	Relative risk	Assessment	Adjustment
	on		(%)		-up,y	(cases)	Assessment	(high vs. low intake)		of T2DM	
Sluijs,2012	(EPIC-	8 countries	50%	52	16	24,475	FFQ	Total dairy (628.9 g vs.79.7 g)	0.97 (0.82,1.15)	Self reporting, primary	Center, age, sex, BMI, educational
	InterAct)	in Europe				(10,694)	24-h dietary	Milk (486.1 g vs. 0.3 g)	1.08 (0.90,1.31)	care registers,	level, smoking, physical activity,
							recall data	Yogurt (190.4 g vs. 8.4 g)	0.89 (0.77,1.03)	secondary care	alcohol intake, fruit plus vegetables,
								Cheese (73.7 g vs. 3.2 g)	0.83 (0.7,0.98)	registers, medication	red meat, processed meat, sugar
										use(drug registers),	
										hospital admissions,	
								Fermented dairy (220.7 g vs. 40.4 g)	0.85 (0.73,0.99)	and mortality data	sweetened soft drinks, coffee, cereals,
Grantham,	AusDiab	Australia	45%	52	5	5,582	121-item FFQ	Total dairy (408 g vs. 346 g)	0.71 (0.48,1.05)	75 g OGTT	Age, sex, energy intake, family
2012						(209)		Low-fat milk (375 g vs. 200 g)	0.65 (0.44,0.94)		history of diabetes, education level,
								Full-fat milk (375 g vs. 200 g)	1.18 (0.78,1.79)		level of physical activity, smoking,
								Yogurt (73 g vs. 3 g)	1.14 (0.78,1.67)		TAG, HDL cholesterol, systolic blood
								Cheese (20 g vs. 6 g)	0.78 (0.48,1.15)		pressure, waist circumference and hip
Louie, 2012	BMES	Australia	42%	63.5	10	1,824	145-item FFQ	Total dairy (3.1 vs. 0.5)	1.50 (0.47,4.77)	Self-reporting, taking	Age, sex, smoking, physical activity,
						(145)		Low-fat dairy (2.1 vs. 0)	1.09 (0.57,2.09)	medication for T2DM,	dietary glycemic load, fibre, total
								Full-fat dairy (1.9 vs. 0.1)	0.87(0.48,1.58)	fasting blood glucose	energy intake and family history of
										>7.0 mmol/L	type 2 diabetes, calcium.
Struijk,	Inter99	Denmark	47.5%	30–60	5	5,232	FFQ	Total dairy (578 g vs. 47 g)	0.96 (0.58,1.58)	75 g OGTT	Age, gender and intervention group,
2012						(214)		Low-fat dairy (536 g vs. 57 g)	0.85 (0.52,1.40)		diabetes family history, education
								Full-fat dairy (89 g vs. 4 g)	0.94 (0.56,1.58)		level, physical activity smoking
								Milk (546 g vs. 16 g)	0.95 (0.58,1.57)		status, alcohol intake, wholegrain
								Cheese (49 g vs. 4 g)	0.78 (0.47,1.29)		cereal, meat, fish, coffee, tea, fruit,
								Fermented dairy (260 g vs. 13 g)	0.86 (0.50,1.47)		vegetables, energy intake, change in
											diet waist circumference
Soedamah­	Whitehall II	England	72%	56	9.8	4,186	114-item FFQ	Total dairy (575 g vs. 246 g)	1.30 (0.95,1.77)	Self-reporting, and	Age, ethnicity and employment grade,
Muthu, 2012						(273)		Low-fat dairy (458 g vs. 28 g)	0.98 (0.73,1.31)	75 g OGTT	smoking, alcohol intake, BMI,
								Full-fat dairy (182 g vs. 27 g)	1.23 (0.91,1.67)		physical activity and family history of
								Yogurt (117 g vs. 0 g)	1.04 (0.87,1.58)		CHD/hypertension, fruit and
								Milk (441 g vs. 147 g)	0.97 (0.71,1.31)		vegetables, bread, meat, fish, coffee,
								Fermented dairy (105 g vs. 17 g)	1.17 (0.87,1.58)		tea and total energy intake.
								Cheese (31 g vs. 6 g)	1.20 (0.88,1.64)		
Margolis,	WHI-OS	USA	0	50–79	8	82,076	122-item FFQ	Total dairy (3.4 vs.0.5)	0.93 (0.83,1.04)	Self-reporting	Age, race/ethnicity, total energy
2011						(3946)		Low-fat dairy (2.8 vs.0.05)	0.65 (0.44,0.96)	confirmed by review	intake, income, education, smoking,
								Full-fat dairy (1.3 vs.0.06)	0.80 (0.65,0.99)	of medical records	alcohol intake, family history of
								Yogurt (≥2/wk vs.<1/mo)	0.46 (0.31,0.68)		diabetes, postmenopausal hormone
											therapy, blood pressure, BMI,
											physical activity, dietary glycemic
											load, dietary total fat, dietary total
											fiber, magnesium
Malik, 2011	NHS II cohort	USA	0	34–53	8	37,038	133-item FFQ	Total dairy (2.14 vs. 0.62)	0.75 (0.55,1.02)	self-reporting	Age, BMI, total energy intake, family
						(550)		Low-fat dairy (1.44 vs.0.18)	0.74 (0.54,1.01)	confirmed by	history of diabetes, smoking, physical
								Full-fat dairy (1.14 vs. 0.19)	0.72 (0.53,0.99)	review of medical	activity, alcohol, oral contraceptive
										records	use, hormone replacement therapy.
											Polyunsaturated, saturated fat, glycemic
											load, fiber, trans fat, processed meat,
											carbonated soft drinks, fruit drinks,coffee
Kirii, 2009	JPHC cohort	Japan	57%	40–69	5	59,796	FFQ	Total dairy (≥300 g vs. <50 g)	Males 1.18 (0.90,1.56)	Self-reporting,	Age, area, BMI, family history of
						(1,114)			Females 0.71(0.51,0.98)	Validity verified by	diabetes mellitus, smoking, alcohol
								Milk (≥200 g vs. <50 g)	Males 1.02(0.85,1.24)	medical record data	intake hypertension, exercise, coffee,
									Females 0.87(0.70,1.09)	and plasma glucose	magnesium, total energy
								Cheese (≥5 g vs. 0 g)	Males 0.88 (0.64,1.21)	random samples.	
									Females 1.12(0.80,1.57)		
								Yogurt (≥60 g vs. 0 g)	Males 1.01 (0.75,1.36)		
									Females 0.77(0.58,1.01)		
Villegas,	SWHS cohort	China	0	51	6.9	64,191	FFQ	Milk (250 vs. 0)	0.60 (0.41,0.88)	Self-reporting,	Age, energy intake, BMI, waist-hip
2009						(2,270)				fasting glucose and	ratio, smoking status, alcohol
										OGTT	consumption, physical activity,
											income level, education level,
											occupation, and hypertension.
Elwood	Caerphilly	UK	100	45–59	20	640	FFQ and 7-	Milk	0.57 (0.20,1.63)	Self-reporting	Age, smoking, BMI and social class
2007	prospective					(41)	day weighed				
	study						intake				
Liu, 2006	WHS cohort	USA	0	55	10	37,183	131-item FFQ	Total dairy (>2.9 vs. <0.85)	0.68 (0.52,0.89)	Diagnostic criteria	Age, total energy intake,
						(1063)		Low-fat dairy (>2.0 vs. ≤0.27)	0.69 (0.522,0.91)	of ADA, based on	randomized-treatment assignment,
								Full-fat dairy (>1.33 vs. <0.2)	0.99 (0.82,1.20)	self-reporting, 3	family history of diabetes, smoking,
								Yogurt (≥2/wk vs.<1/mo)	0.82 (0.70,0.97)	complementary	BMI, hypercholesterolemia,
								Whole milk (≥2/wk vs.<1/mo)	1.04 (0.84,1.30)	approaches to	hypertension, physical activity
								Skim milk (≥2/wk vs.<1/mo)	0.92 (0.78,1.09)	validate the cases	hormones, alcohol consumption, fiber,
								Cottage cheese (≥2/wk vs<1/mo)	0.86 (0.71,1.05)		total fat, and dietary glycemic load,
								Ice cream (≥2/wk vs.<1/mo)	0.88 (0.74,1.05)		calcium, vitamin D, and magnesium.
								Other cheese (≥2/wk vs.<1/mo)	0.80 (0.64,1.01)		
Van Dam,	Black Women’s	USA	0	21–69	8	41,186	FFQ	Total dairy (2.53 vs. 0.07)	0.93 (0.75,1.15)	Self-reporting,	Age, total energy intake, BMI,
2006	Health Study					(1,964)		Low-fat dairy (1.22 vs. 0)	0.87 (0.76,1.00)	validity verification	smoking physical activity, alcohol,
								Full-fat dairy (1.33 vs. 0.07)	1.03 (0.88,1.20)	of a random sample	family history of diabetes, education
											level, coffee, sugar-sweetened soft
											drink, processed meat, red meat,
										.	calcium or magnesium intake
Pittas, 2006	NHS cohort	US	0	30–55	20	83,779	FFQ	Total dairy (3.9 vs. 0.9)	0.79 (0.70,0.90)	Criteria by	Age, BMI, hypertension, family
						(4,843)				National Diabetes	history of diabetes, smoking, physical
										Data Group and	activity, caffeine, alcohol, and state of
										ADA.self-reporting	residence, fat (saturated,
											polyunsaturated, or trans), cereal fiber,
											glycemic load, magnesium, and retinol
Choi, 2005	HPFS cohort	USA	100	43–75	12	41,254	FFQ	Total dairy (≥2.9 vs. <0.9)	0.75 (0.61,0.93)	Criteria by	Age, total energy intake, family history
						(1243)		Low-fat dairy (>1.58 vs. <0.14)	0.74 (0.60,0.91)	National Diabetes	of diabetes, smoking, BMI,
								Full-fat dairy (>1.72 vs.<0.38)	0.82 (0.66,1.02)	Data Group. Based	hypercholesterolemia, hypertension,
								Yogurt (≥2/wk vs. <1/mo)	0.83 (0.66,1.06)	on self-reporting.	physical activity, alcohol, fiber,
								Whole milk (≥2/wk vs. <1/mo)	1.19 (1.00,1.43)	Validity verified	trans-fat polyunsaturated to saturated
								Low-Fat milk (≥2/wk vs. <1/mo)	0.78 (0.63,0.97)	with medical	fat, glycemic load
								Cottage cheese (≥2/wk vs. <1/mo)	0.96 (0.80,1.17)	records in a sample	
								Other cheese (≥2/wk vs. <1/mo)	0.88 (0.67,1.16)	of 71 participants.	
								Ice cream (≥2/wk vs. <1/mo)	0.78 (0.64,0.95)		
Montonen,	Finnish Mobile	Finland	50	40–69	23	4304	dietary history	Regular dairy (>305 vs. <39)	0.81(0.62–1.08)	from the Social	Adjusted for age, sex, body mass
2005	Clinic Health					(383)	interview	Low fat dairy (>0 vs. 0)	0.90(0.60,1.36)	Insurance	index, energy intake, smoking, family
	Examination							Whole milk (>878 vs. <326)	1.06(0.75,1.50)	Institution’s	history of diabetes, and geographic
	Survey									nationwide register	area
										of persons	
										receiving drug	
										reimbursement	
Ericson,	Malmö Diet and	Sweden		57	8	23 531	148-FFQ	Total dairy women (6.0 vs. 1.8)	0·88 (0·70, 1·09)	Self report, and	sex, smoking status, alcohol
2013	Cancer cohort					(837)		Total dairy men (6.3 vs. 1.8)	1·20 (0·98, 1·47)	verified with an	consumption, leisure-time physical
										inquiry to the	activity, BMI, waist-to-hip ratio,
										treating physician,	hypertension, history of high blood lipid
										local cancer	levels at baseline, education, vitamin
										registries	supplementation, non-consumption of the
											respective food group, total energy intake
											(kJ/day).

FFQ, food-frequency questionnaire; OGGT, oral glucose tolerance test; BMI, body mass index TAG,triglyceride; HDL, High density lipoproteins; ADA, American Diabetes Association CHD, coronary heart disease; EPIC-InterAct European Prospective Investigation into Cancer and Nutrition cohort; AusDiab, Australian Diabetes Obesity and Lifestyle Study; BMES, Blue Mountains Eye Study; WHI-OS, Women’s Health Initiative observational study; NHS, The Nurses’ Health Study; JPHC, Japan Public Health Center-based Prospective Study; SWHS, Shanghai Women’s Health Study; WHS, Women’s Health Study; HPFS, Health Professionals Follow-up Study.

### Total Dairy Products Intake and T2DM Risk

In all, 13 studies [10], [11], [12], [13], [14], [15], [16], [17], [18], [19], [20], [23], [34] including 457,893 subjects (27,095 cases) were analyzed.

In all, 13 studies(8–18; 21) including 457,893 subjects (27,095 cases) were analyzed.

#### High versus low intake

**Figure 3:**
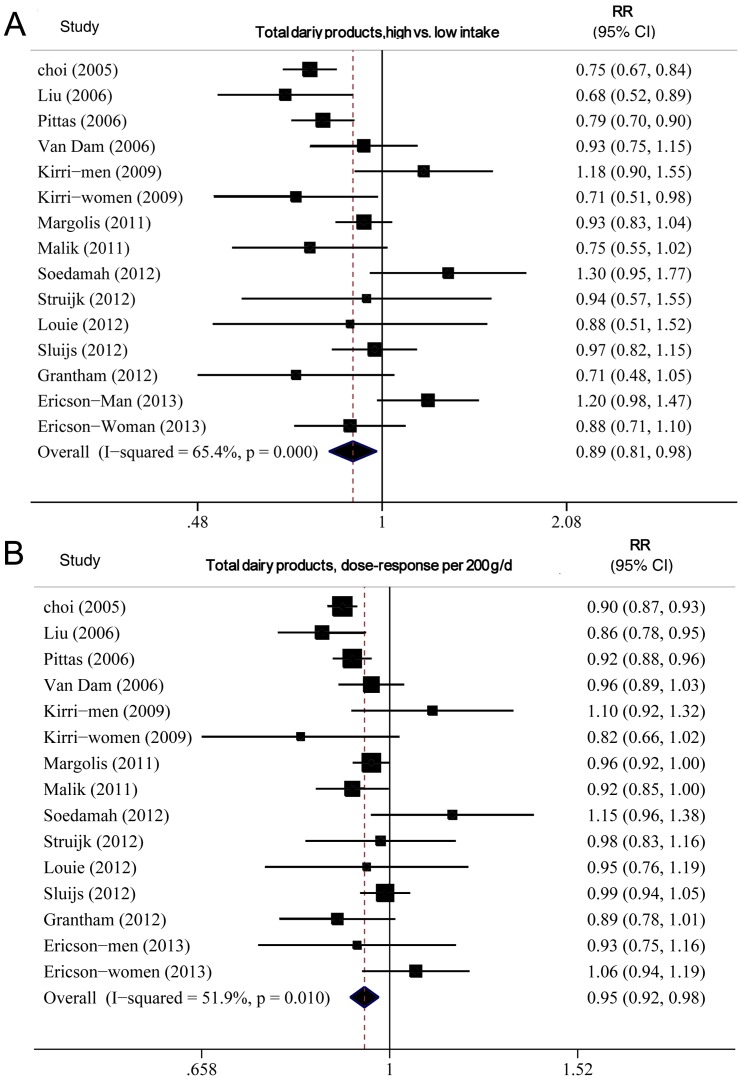
Forest plot of relative risk (RR) for total dairy products intake and T2DM. A, highest versus lowest intake. B, dose–response analysis (200 g/d). Weights are from random effects analysis.

The summary RR for all studies was 0.89 (95% CI 0.81–0.98), with moderate heterogeneity, I^2^ = 65.4% and P_heterogeneity_ = 0.000 (Figure 3A).

#### Dose–response analysis

**Figure 4:**
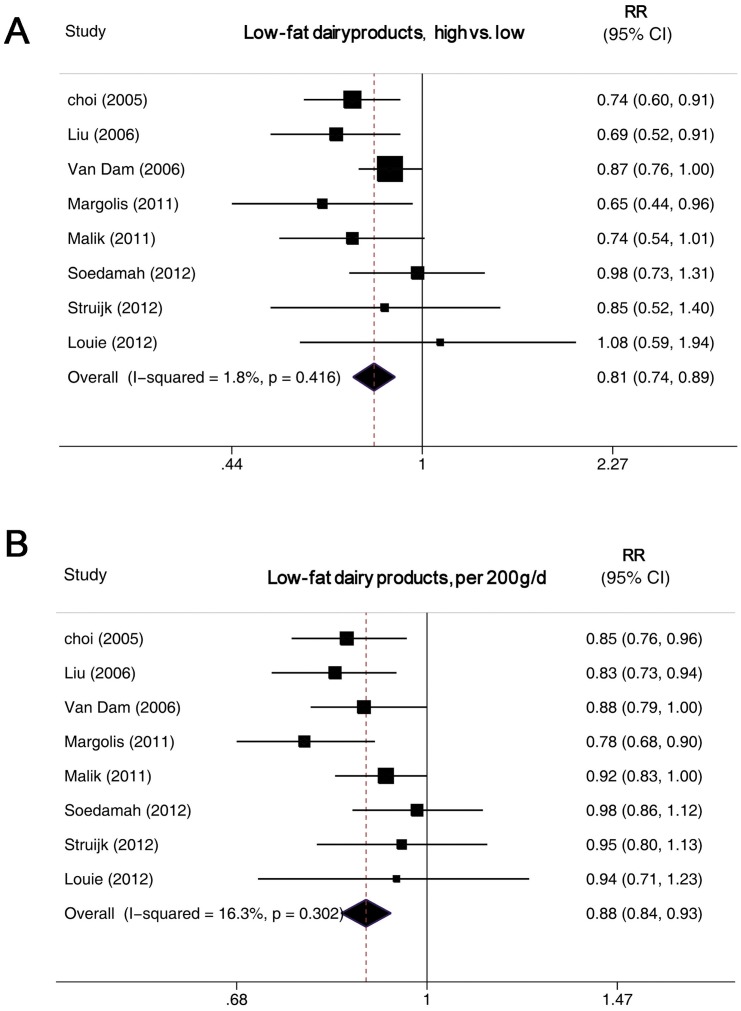
Dairy products and incidence of T2DM, nonlinear dose–response analysis. A, total dairy. B, low-fat dairy.

The summary RR for an increase of 200 g/day was 0.94 (0.91–0.97), with moderate heterogeneity, I^2^  = 51.6%, P_heterogeneity_  = 0.02 (Figure 3B). On subgroup analysis (Table 2), we found an inverse association of total dairy intake and T2DM risk in all strata except European studies and studies not adjusting for family history of T2DM, although in some analyses the associations were not statistically significant. None of the results differed significantly by sex (P = 0.21 for all comparisons). On univariate meta-regression analysis, geographic location, adjustment for family T2DM history, and glycemic load were significant predictors of the heterogeneity (p = 0.05, p = 0.04 and p = 0.04, respectively). But on multivariate meta-regression, we failed to identify the source of heterogeneity. We found no evidence of publication bias by Egger's test (P = 0.37), Begg's test (P = 0.58) or funnel plot(see Appendix Figure 1). We also found a nonlinear association of total dairy product intake and T2DM risk, P_for nonlinearity_ <0.001, with most of the risk reduction occurring with intake up to about 200 g/d; higher intake were associated with a further but more modest decrease in risk (Figure 4A).

**Table 2:** Subgroup analyses of total and low-faty dairy products intake and T2DM, dose–response analysis.

			Total dairy						Low-fat dairy					
			n	RR (95% CI)	I^2^ (%)	P_a_	P_b_	P_c_	n	RR (95% CI)	I^2^ (%)	P_a_	P_b_	P_c_
**All studies**			12	0.94 (0.91,0.97)	51.6	0.02			8	0.88 (0.84,0.93)	16.3	0.30		
**Duration**														
	<10		6	0.95 (0.92,0.98)	4.3	0.39			4	0.88 (0.82,0.95)	32.5	0.21		
	≥10		6	0.94 (0.89,0.99)	68.6	0.01	0.65		4	0.89 (0.82,0.96)	23.5	0.27	0.93	
**Sex**														
	Female		6	0.93 (0.90,0.96)	26.9	0.23			4	0.86 (0.80,0.92)	29.6	0.23		
	Male		2	0.97 (0.80,1.18)	78.2	0.03			1	0.85 (0.76,0.96)				
	Both		5	0.98 (0.92,1.05)	24.5	0.26	0.21		3	0.94 (0.71,1.23)	0	0.94	0.16	
**Geographic location**														
	United States		6	0.92 (0.90,0.95)	42.7	0.12			5	0.86 (0.82,0.91)	8	0.36		
	Europe		3	1.01 (0.94,1.08)	18.6	0.29			2	0.97 (0.87,1.06)	0	0.94	0.10	0.57
	Asia		1	0.96 (0.72,1.28)	75.5	0.04			1	0.94 (0.71,1.24)				
	Australia		2	0.90 (0.81,1.01)	0	0.62	0.04	0.08						
**No. of cases**														
	<500		4	0.98 (0.88,1.10)	41.5	0.16			3	0.97 (0.88,1.06)	0	0.94		
	500–1500		4	0.91 (0.86,0.95)	38.5	0.17			3	0.88 (0.82,0.93)	0.9	0.37		
	≥1500		4	0.95 (0.92,0.98)	32.7	0.22	0.43		3	0.83 (0.74,0.94)	40	0.20	0.10	
**Study type**														
	Prospective		11	0.93 (0.90,0.96)	44.7	0.05			8	0.88 (0.84,0.93)	16.3	0.30		
	Case cohort		1	0.99 (0.94,1.05)			0.22							
**Adjustment method**														
	COX		8	0.94 (0.91,0.97)	63.9	0.01				0.88 (0.82,0.93)	32.6	0.19		
	Logistic		4	0.94 (0.86,1.04)	24.8	0.26	0.92			0.95 (0.82,1.10)	0	0.95	0.39	
**Adjustment factors**														
BMI		Yes	9	0.94 (0.91,0.97)	53	0.02			6	0.87 (0.83,0.92)	8	0.37		
		No	3	0.98 (0.84,1.16)	60.9	0.02	0.57		2	0.97 (0.86,1.10)	2	0.79	0.14	0.45
Diabetes history		Yes	1	0.93 (0.90,0.95)	30.2	0.16			1	0.87 (0.83,0.92)	0	0.45		
		No	2	1.04 (0.91,1.19)	58.2	0.12	0.048	0.24	0	0.98 (0.86,1.12)			0.16	
Glycemic load		Yes	6	0.92 (0.89,0.95)	33.7	0.18			5	0.86 (0.81,0.92)	13.3	0.33		
		No	6	0.98 (0.92,1.03)	37.7	0.14	0.067	0.41	3	0.93 (0.86,1.00)	0	0.47	0.20	
Fat		Yes	5	0.92 (0.89,0.95)	46.4	0.11			4	0.85 (0.80,0.92)	28.9	0.24		
		No	7	0.98 (0.93,1.03)	27.7	0.21	0.067	0.56	4	0.93 (0.86,1.00)	0	0.68	0.17	
Fiber intake		Yes	5	0.92 (0.89,0.95)	46.4	0.11			4	0.85 (0.80,0.92)	28.9	0.24		
		No	7	0.94 (0.91,0.97)	27.7	0.21	0.067		4	0.93 (0.86,1.00)	0	0.68	0.17	
Coffee		Yes	6	0.98 (0.93,1.03)	38	0.14			4	0.83 (0.77,0.89)	0	0.64		
		No	6	0.92 (0.89,0.95)	38	0.17	0.069	0.47	4	0.92 (0.87,0.98)	0	0.68	0.06	0.41
Fruit, vegetables		Yes	9	0.93 (0.90,0.96)	37.1	0.11			2	0.94 (0.87,1.01)	0	0.55		
		No	3	0.99 (0.90,1.08)	63.1	0.07	0.17		6	0.85 (0.81,0.91)	0	0.44	0.1	
Meat		Yes	7	0.92 (0.89,0.95)	44.5	0.08			4	0.83 (0.77,0.89)	0	0.64		
		No	5	0.97 (0.93,1.02)	28.4	0.23	0.098	0.58	4	0.92 (0.87,0.98)	0	0.68	0.06	0.34
Cacium, magnesium		Yes	7	0.95 (0.91,0.98)	44.5	0.08			4	0.84 (0.78,0.90)	0	0.50		
		No	5	0.93 (0.88,0.99)	48.4	0.10	0.50		4	0.92 (0.86,0.97)	0	0.43	0.12	
Energy intake		Yes	9	0.93 (0.90,0.96)	43.3	0.08			7	0.88 (0.83,0.94)	28.2	0.21		
		No	3	0.98 (0.92,1.04)	33.6	0.21	0.13		1	0.88 (0.78,0.99)			0.95	

BMI, body mass index; n, the number of studies,P_a_, for heterogeneity within each subgroup; P_b_, for heterogeneity between subgroups with univariate meta-regression analysis; P_c,_ for heterogeneity with multivariate meta-regression analysis.

### Low- and Full-fat Dairy Intake and T2DM Risk

8 studies [10], [11], [12], [13], [14], [15], [16], [23] including 260,700 subjects (9,398 cases) were analyzed.

#### High versus low intake

**Figure 5:**
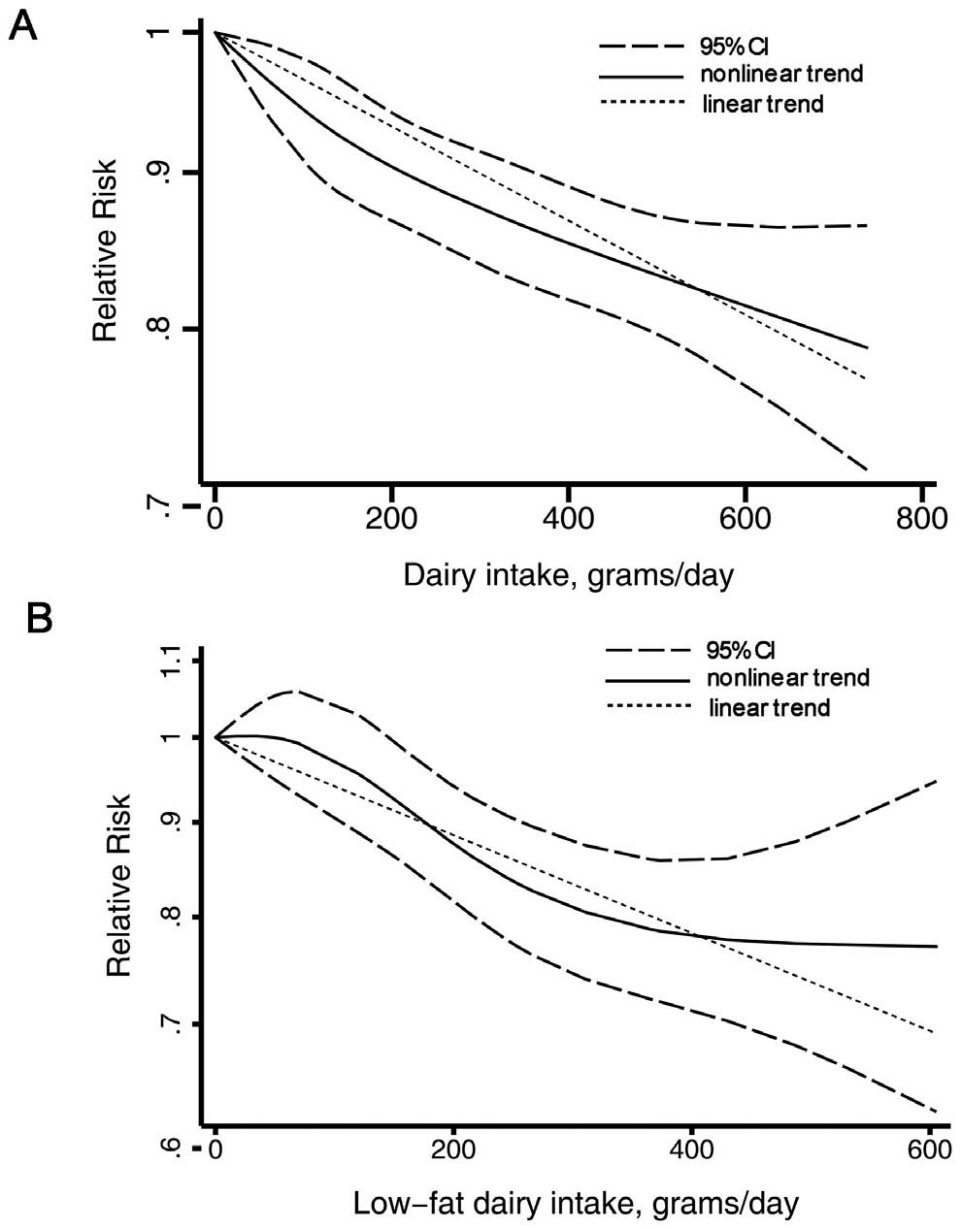
Forest plot of RR for low-fat dairy products intake and T2DM. A, highest versus lowest intake. B, dose–response analysis (200 g/d). Weights are from random-effects analysis.

**Figure 6:**
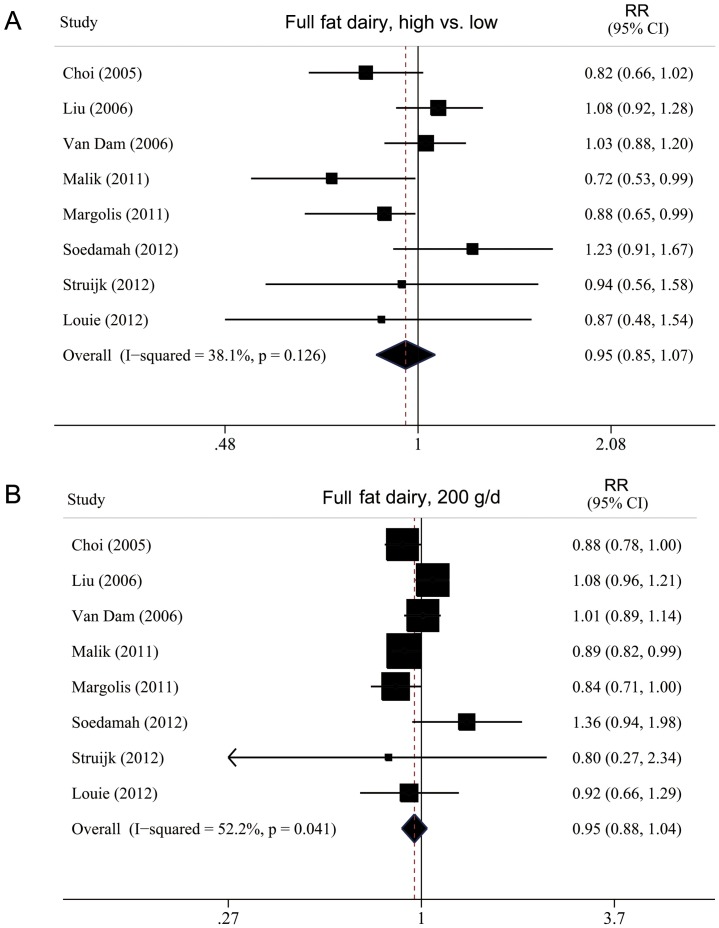
Forest plot of RR for full-fat dairy products intake and T2DM. A, highest versus lowest intake. B, dose–response analysis (200 g/d). Weights are from random-effects analysis.

Low-fat dairy consumption was inversely associated with T2DM risk, with a pooled RR of 0.81 (0.74–0.89) (Figure 5A). Full-fat dairy consumption was not associated with T2DM risk, with a summary RR of 0.95 (0.85–1.07) (Figure 6A). We found no significant heterogeneity for the associations of low-fat (I^2^ = 1.8%; P_heterogeneity_  = 0.42) or full-fat dairy consumption (I^2^ = 38.1%; P_heterogeneity_  = 0.13).

#### Dose–response analysis

The summary RR for a 200-g/day increase in low-fat dairy intake was 0.88 (0.84–0.93), with no evidence of heterogeneity, I^2^ = 16.3% and P_heterogene_ity  = 0.32 (Figure 5B). The summary RR for a 200-g/day increase in full-fat dairy intake was 0.95 (0.88–1.04), with evidence of heterogeneity, I^2^ = 52.2% and P_heterogeneity_  = 0.04 (Figure 6B). We found an inverse association of low-fat dairy intake and T2DM risk for all strata, although in some analyses the associations were not statistically significant (Table 2). On univariate meta-regression analysis, the effect was weaker, although not significantly, for studies with than without adjustment for coffee and meat intake (P = 0.06 for both). But on multivariate meta-regression, we failed to identify the source of heterogeneity. On We found a nonlinear association of low-fat dairy intake and T2DM risk, P_for nonlinearity_  = 0.02, with most of the risk reduction occurring with intake up to about 300 g/day; higher intake (>400 g/day) was not associated with a further decrease in risk (Figure 4B).

### Milk Intake and T2DM Risk

#### High versus low analysis

**Figure 7:**
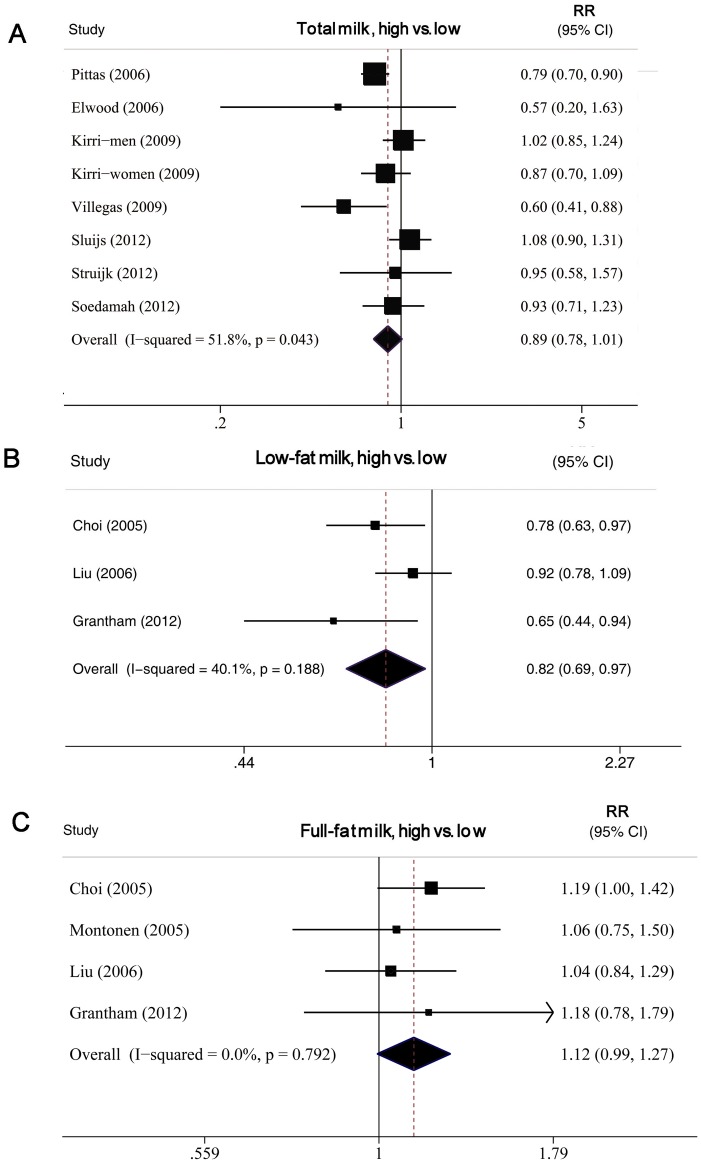
Forest plot of RR for highest versus lowest milk intake and T2DM. A, total milk. B, low fat milk. C, full fat milk. Weights are from random-effects analysis.

9 studies [11], [13], [15], [17], [18], [19], [20], [22], [23] including 327,039 subjects (21,755 cases) were analyzed. For 6 studies [11], [17], [18], [19], [22], [23], data were available on the association of total milk intake and T2DM risk, for 3 studies [13], [15], [20], milk consumption was analyzed as full-fat milk (or whole milk) and low-fat milk (or skim milk) and for 1 study, data was reported as full fat milk only [35]. The summary RR for total milk intake was 0.89 (0.78–1.01), with moderate heterogeneity, I^2^ = 51.8% and P _heterogeneity_ = 0.043. The summary RR for low-fat and full-fat milk intake was 0.82 (0.69–0.97, I^2^ = 40% and P_heterogeneity_ = 0.19) and 1.12 (0.99–1.27, I^2^ = 0% and P_heterogeneity_ = 0.79), respectively (Figure 7).

#### Dose–response analysis

8 studies [11], [13], [15], [17], [18], [19], [20], [22], [23] were analyzed. The summary RR for a 200-g/day increase in total milk intake was 0.89 (0.79–1.01), with evidence of moderate heterogeneity, I^2^ = 66.3% and P_heterogeneity_  = 0.005 (data not shown). The summary RR for a 200-g/day increase in full- and low-fat milk intake was 1.27 (0.97–1.67, I^2^ = 0% and P_heterogeneity_ = 0.58) and 0.83 (0.70–1.00, I^2^ = 14% and P_heterogeneity_ = 0.21), respectively.

### Yogurt and Cheese Intake and T2DM Risk

Seven studies [11], [12], [13], [15], [17], [19], [20] including 254,552 subjects (18,532 cases) were analyzed for yogurt intake. Seven studies [11], [13], [15], [17], [19], [20], [23] including 178,429 subjects (14,810 cases) were analyzed for cheese intake.

#### High versus low intake

Yogurt and cheese intake were inversely associated with T2DM risk. The pooled RRs were 0.85 (0.75–0.97, I^2^ = 55% and P_heterogeneity_  = 0.02) and 0.82 (0.77–0.87, I^2^ = 0% and P_heterogeneity_  = 0.82), respectively.

#### Dose–response analysis

Yogurt and cheese intake were inversely associated with T2DM incidence. The pooled RRs were 0.91 (0.82–1.00, I^2^ = 74%, P_heterogeneity_ = 0.001) per 50 g/d and 0.80 (0.69–0.93, I^2^ = 59%, P_heterogeneity_ = 0.02) per 30 g/d, respectively.

### Other dairy products

Intake of other types of dairy products except ice cream (n = 2 studies) were not significantly associated with T2DM risk (0.84 [0.73–0.95]). The pooled RR for total fermented dairy intake (n = 3 studies) was 0.94 (0.75–1.18) and cream (n = 2 studies) was 0.96 (0.84–1.12).

## Discussion

This meta-analysis showed that total dairy intake was associated with a 6% lower risk of T2DM per 200 g/day consumption. When examining different types of dairy products in relation to T2DM risk, we found significant inverse associations of intake of low-fat dairy, low-fat milk, cheese and yogurt and T2DM. We found no association of intake of full-fat dairy as well as total and full-fat milk and T2DM. We also clarified a nonlinear association of both total and low-fat dairy intake and incidence of T2DM.

The hypothesis that dairy products intake protects against T2DM has received much interest among medical professionals and the general population. In intervention studies, the Dietary Approaches to Stop Hypertension (DASH) diet (a dietary pattern focusing on low-fat milk and other dairy products) increased high-density lipoprotein levels, reduced triglycerides levels, reduced blood pressure (both systolic and diastolic), contributed to weight loss, and reduced fasting blood glucose in both men and women as compared with the control diet [36]. In epidemiological studies, the association of dairy products intake and T2DM has been explored with inconsistent results [37].

Our findings for high versus low dairy intake are consistent with results from previous meta-analyses [24], which only included 7 studies. High versus low analyses are limited because true differences in the level and range of intake between studies are not considered and may contribute to heterogeneity in the results. With the accumulated evidence, we were able to enhance the precision of the risk estimates, perform dose–response analyses of different dairy products and explore the shape of the dose–response curve and sources of heterogeneity, thereby increasing the clinical relevance of our findings [38].

In addition, the presence of both linear and nonlinear dose–response relationships of specific dairy products strengthened the findings of an association of dairy products intake and risk of T2DM. In the linear dose-response analysis, we found a 6% and 12% lower risk of T2DM per 200 g/day intake of total and low-fat dairy products, respectively. Furthermore, we discovered a potential nonlinear association of total and low-fat dairy products intake and T2DM. A low threshold of 200 g/day total dairy and 300 g/day low fat dairy may reduce the risk by about 10% or 15% respectively. Intake above that level seems to have further but modest additional benefit for T2DM risk.

Dairy is a major source of dietary calcium and magnesium, 2 minerals that have a role in the development of T2DM, for potential in improving pancreatic B-cell function and insulin sensitivity [39]. Experimental [39], prospective cohort studies [40], [41] and a recent meta-analysis [42] have provided convincing evidence to support the direct effects of calcium and magnesium intake on insulin resistance and T2DM. In this study, we found that the association of dairy intake and T2DM risk remained unchanged after adjusting for diet calcium and/or magnesium (7 studies), so other major components in dairy products could account for the association. Recently, the beneficial physiological effects of dairy protein, such as whey protein, on the control of food intake and glucose metabolism have been reported. Studies have shown the insulinotropic and glucose-lowering properties of whey protein in healthy and T2DM subjects [43]. Furthermore, in addition to milk proteins, trans-palmitoleate, obtained primarily from dairy intake, is associated with reduced incidence of diabetes [9].

Our analysis of high- and low-fat dairy products revealed an inverse association of only low-fat dairy food intake and T2DM risk. This support the present recommendations by health authorities and governments to eat low-fat rather than full-fat dairy products [44]. We think the most prominent relationship was from residual confounding by factors related to a more unhealthy diet or lifestyle. On the other hand, we can not rule out the association between the intake of saturated fatty acid (SFA). Dairy products contributed to 15% of the total dietary SFA intake [45]. Although prospective cohorts demonstrate no significant association between SFA intake and risk of T2DM, some findings from experimental and observational studies have showed that SFA intake was inversely associated with insulin sensitivity [45], [46], [47]. Finally, the likelihood of publication bias effects may cause uncertain results. For analysis of the milk products, only 3 of 14 studies separately evaluated whole vs. low-fat milk, and thus it seems that publication bias could account for the observed difference between low vs. whole fat milk. Furthermore, because cheese, even low-fat cheese, has higher fat and saturated fat than whole milk yet was still associated with lower risk, it appears less likely that the observed difference between whole fat and low fat milk would be due to higher fat or saturated fat content in whole milk. Further confirmatory results of appropriately powered studies are still needed.

Cheese, which has far more fat than whole-fat milk, more than half of which is saturated Evidence suggests that saturated fat intake has an adverse effect on insulin sensitivity and increases the risk of T2DM. In our analysis, we found inverse association between both cheese and yogurt intake and incidence of T2DM. The exact mechanisms responsible for the significant inverse association between cheese and yogurt and T2DM are unknown. It could be partly explained by the fact that both dairy subgroups are a good source for vitamin K2. Vitamin K2 is exclusively synthesized by bacteria and is therefore only present in fermented dairy products such as cheese and yogurt due to the bacterial starter fermentation [48]. Vitamin K2 has recently been linked to a reduced risk of T2DM [49]. Additionally, these dairy subcategories are particularly high in the fat-soluble vitamin D, which has been found to be inversely associated with T2DM [50], [51].

We did not find a consistent pattern of difference or heterogeneity in results by sex or any other study characteristics examined, except for geographic location, which significantly modified the association between total dairy products intake and T2DM risk. We found a significant inverse association among US studies, with no evidence of a protective effect of total dairy food intake in European or Asia studies. This may be a chance finding, because only 3 European studies and 1 Asian study were included in this subgroup analysis or could be due to other factors. As well, differences in the ranges of intake or intake in the referent category could explain these results. Because of the nonlinear association between total dairy food intake and T2DM risk with the strongest reduction at low levels of intake, some studies may have missed an effect because the intake in the referent category may have been already sufficient to reduce risk. For example, in some European studies, intake of total dairy food in the referent category was >200 g/d but was <200 g/d for all US studies. As well, types of dairy food intake may vary between populations. In addition, differences in study size and follow-up time may contribute to the variations. Further cohort studies of specific dairy products and T2DM risk in different populations are needed.

Our meta-analysis contains some limitations. Publication bias is a major concern for analyses that depend on only a few studies. For example, in our analysis, only 4 of the 15 studies separately evaluated full or low-fat milk. So the efficiency of analysis on different milk production was limited. The inverse association we found between dairy products intake and T2DM risk could be due to unmeasured or residual confounding. Higher intake of dairy products, especially low-fat dairy products, is often associated with other lifestyle factors, including increased physical activity, low prevalence of smoking, and overweight/obesity, although different types of dairy products may be differentially associated with some of these confounders. In addition, the results were generally similar in the subgroup analyses when we stratified results by adjustment for confounding factors or other study characteristics, with no heterogeneity between subgroups for total and low-fat dairy product consumption. Only the analysis of total dairy products revealed some indication of heterogeneity, with studies that adjusted for family history of T2DM showing an inverse association with T2DM; studies that did not adjust for family history of T2DM showed a nonsignificant positive association, which suggests potential confounding.

Measurement errors in the assessment of dietary intake are known to bias effect estimates. Our results are based on data from cohort studies, in which dairy intake was mostly assessed by food-frequency questionnaires. In several studies, validation of the food-frequency questionnaires showed good correlations, of ≈0.6–0.7 for milk or (if not assessed) for protein and calcium, which are good indicators for milk intake. However, we cannot exclude that measurement errors might have resulted in attenuated associations. Dietary changes after baseline can also attenuate associations of dietary intake and T2DM risk; however, only 7 of the included studies [12], [13], [14], [17], [18], [22], [23] used repeated assessments of diet, and the results were similar when using only the baseline questionnaire for the analyses (data not shown). Furthermore, dietary intake data were collected between 1984 and 2003. In earlier studies, full-fat dairy was a major contributor to total dairy intake, whereas in later studies intake was more often low-fat dairy and publication year may have explained the study heterogeneity (p = 0.02). Finally, because all the studies were conducted primarily among middle-aged and older people, these results might not be generalizable to dairy intake in earlier life periods, which might have similar or different effects.

In conclusion, our results suggest a inverse association of intake of dairy products, such as low-fat dairy, cheese and yogurt and T2DM risk. Further cohort studies are warranted to investigate the specific types of dairy products in the association, the impact of measurement errors on estimates, any gender-specific recommendations, and biomarkers of dairy intake.

## Supporting Information

Figure S1 **Funnel plot of assessing evidence of publication bias. A. For total dairy; B. For low-fat dairy; C. For full-fat dairy.**
(TIF)

Figure S2 **Forest plot of RR for highest versus lowest yogurt and cheese intake and T2DM. A, yogurt. B, cheese. Weights are from random-effects analysis.**
(TIF)

Checklist S1 **PRISMA 2009 Checklist**
(DOC)
