# Supplementary figures and images for: Dairy Products Consumption and Risk of Type 2 Diabetes: Systematic Review and Dose-Response Meta-Analysis
Source: PLoS One. 2013 Sep 27;8(9):e73965. doi: 10.1371/journal.pone.0073965 (PMC3785489; doi:10.1371/journal.pone.0073965)

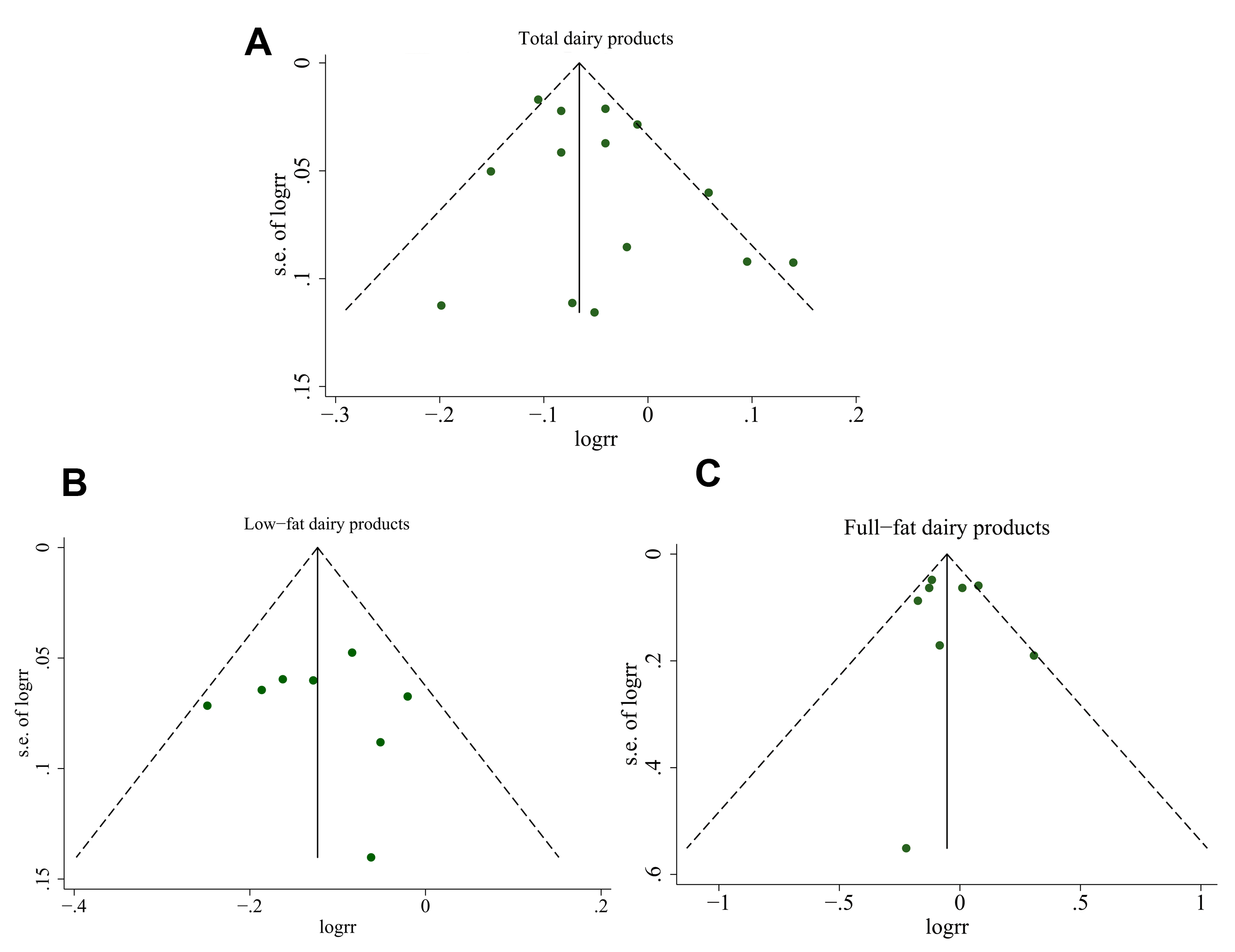

Supplement: Figure S1 — Funnel plot of assessing evidence of publication bias. A. For total dairy; B. For low-fat dairy; C. For full-fat dairy. (TIF) [file pone.0073965.s001.tif]

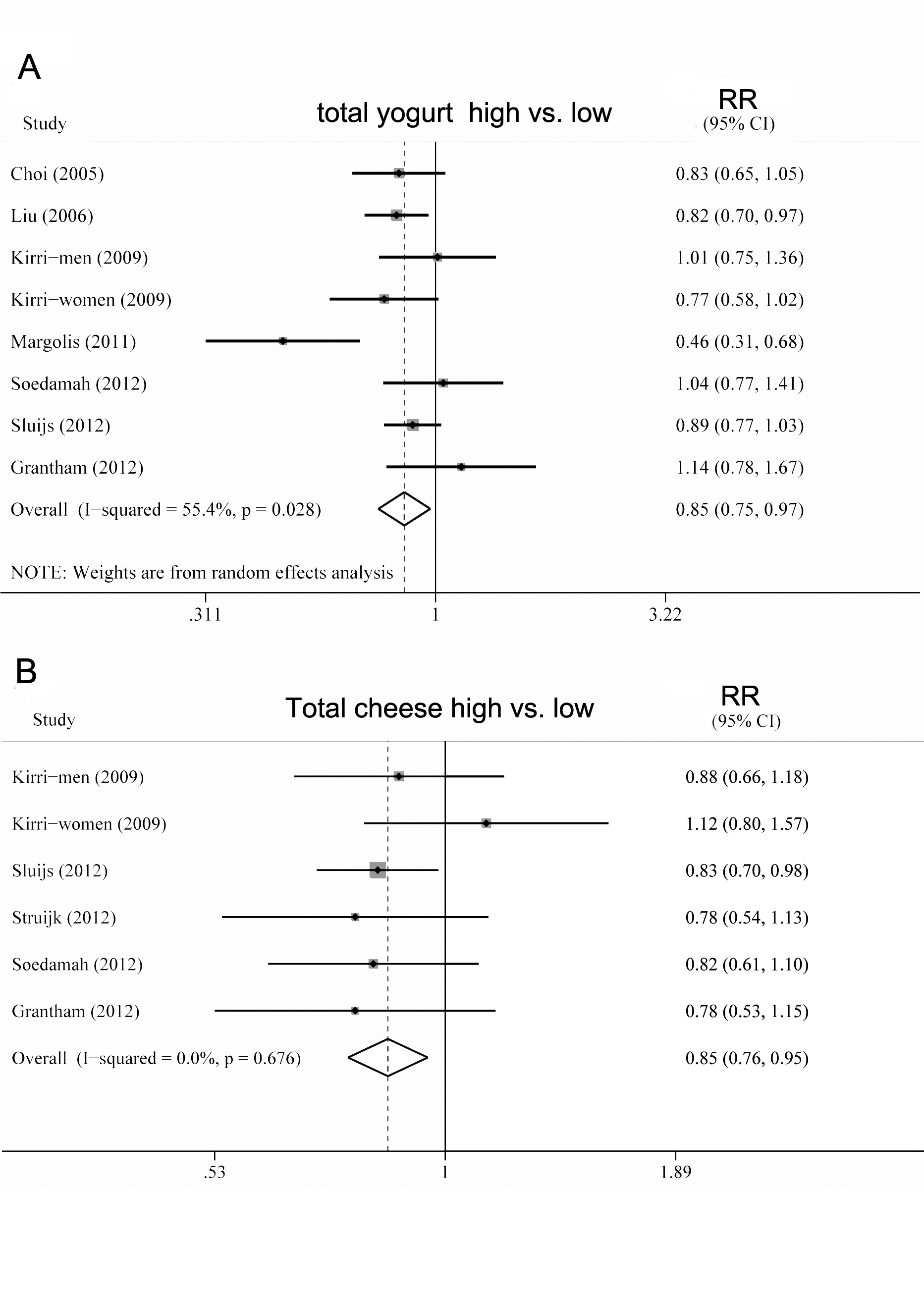

Supplement: Figure S2 — Forest plot of RR for highest versus lowest yogurt and cheese intake and T2DM. A, yogurt. B, cheese. Weights are from random-effects analysis. (TIF) [file pone.0073965.s002.tif]
